# The inflection point: α-Klotho levels and the risk of all-cause mortality

**DOI:** 10.3389/fendo.2025.1405003

**Published:** 2025-03-11

**Authors:** Jianling Song, Hong Li, Xiangdong Fang

**Affiliations:** ^1^ Department of Nephrology, The Second Affiliated Hospital, Jiangxi Medical College, Nanchang University, Nanchang, Jiangxi, China; ^2^ Department of Medical Records, The Second Affiliated Hospital, Jiangxi Medical College, Nanchang University, Nanchang, Jiangxi, China

**Keywords:** α-Klotho, all-cause mortality, NHANES, U-shaped relationship, inflection point

## Abstract

**Purpose:**

The controversial nature of the association between α-Klotho and mortality risk in the general population warrants further investigation. This study aims to examine the correlation between circulating α-Klotho levels and the risk of all-cause mortality

**Methods:**

A sample size of 13,748 individuals from the NAHNES 2005-2016 cycles was included in this study. The effect of different α-Klotho levels (divided into quartiles) on survival was assessed using Kaplan-Meier (KM) curves. Cox proportional hazards models were used to analyze the linear relationship between log α-Klotho and the risk of all-cause mortality. Restricted cubic spline Cox proportional hazards regression model was used to analyze the non-linear relationship between log α-Klotho and risk of all-cause mortality. Threshold effect analysis was performed to determine the most favorable inflection point for log α-Klotho. Stratification and sensitivity analyses were performed to assess the robustness of the results.

**Results:**

A total of 1,569 deaths were reported during the median follow-up period of 5.33 years (2.83-7.83 years). Among the log α-Klotho quartile groups, quartile 1 had the highest mortality rate compared to quartiles 2, 3, and 4. Multifactorial Cox regression analysis revealed a weak association between log α-Klotho and a 44% reduction in the risk of all-cause mortality (p=0.0473). We also found a U-shaped non-linear association between log α-Klotho and risk of all-cause mortality, with an optimal inflection point identified at 2.89 pg/mL. The stability of the U-shaped association between log α-Klotho and mortality risk was observed in various stratification and sensitivity analyses.

**Conclusion:**

This study identified a U-shaped association between circulating α-Klotho levels and risk of all-cause mortality, with a notable inflection point at 2.89 pg/mL. Further investigation is warranted to fully elucidate the potential mechanisms underlying the association between α-Klotho and risk of all-cause mortality in the broader population.

## Introduction

In 1997, Kuro-o (1) and his research group demonstrated that the deletion of the Klotho gene resulted in accelerated aging in mice. It is hypothesized that this gene may have anti-ageing properties. Reduced levels of α-Klotho have been linked to an increased risk of developing a number of diseases. In the context of cardiovascular disease, low levels of Klotho have been associated with a number of adverse outcomes, including myocardial ischemia, cardiac hypertrophy, sinus node dysfunction and sudden cardiac death (2–4). Furthermore, in relation to nephropathic diseases, reduced Klotho has been associated with increased adverse clinical outcomes in patients with chronic kidney disease (CKD), including elevated creatinine levels, CKD progression and CKD-mineral bone disease (5–7). In tumors, Klotho expression is reduced in the majority of tumor samples, and low Klotho expression in tumors is associated with poorer overall survival (8).

Although α-Klotho has been shown to have anti-aging and cardio-protective effects on cardiac and renal function, its association with all-cause mortality has shown different patterns in different populations and disease contexts. Some studies have found that lower α-Klotho levels are associated with a higher risk of mortality in older populations and in patients with CKD (6, 9). However, other studies have observed a U-shaped relationship between α-Klotho levels and the risk of death in CKD populations, where both low and high levels of α-Klotho are associated with a higher risk of death (10). In addition, other studies have failed to find a significant association between α-Klotho levels and mortality risk (11, 12). These contradictory results suggest that the relationship between α-Klotho and mortality risk may be influenced by a variety of factors, and further studies could help to clarify its mechanism of action in the general population.

Given the controversy surrounding the relationship between Klotho and mortality risk, the objective of this study was to re-examine the potential association between Klotho and mortality risk using National Health and Nutrition Examination Survey (NHANES) data.

## Methods

The NHANES is an ongoing research initiative designed to collect demographic data on the nutritional and health status of adults and children in the United States. In order to achieve its stated objectives, the survey employs a stratified, multistage probability sampling design, with the aim of obtaining a sample that accurately represents the US population. The data collection process comprises face-to-face structured interviews conducted in participants’ homes, health screenings conducted at mobile screening centers, and laboratory analysis of biospecimens. The NHANES program has obtained ethical approval from the National Center for Health Statistics Ethics Review Board (https://www.cdc.gov/nchs/nhanes/irba98.htm). This study is in accordance with the Strengthening the Reporting of Observational Studies in Epidemiology (STROBE) guidelines for reporting cross-sectional studies (13).

This study employed a series of continuous data from 2007 to 2016, which included the variables α-Klotho and survival. Furthermore, a number of additional variables were incorporated, including fundamental demographic data (e.g., age, gender, race, education, and economic status), biochemical assessments (e.g., fasting blood glucose (FBG), hemoglobin A1C (HBA1C), total cholesterol (TC), triglycerides (TG), and high-density lipoprotein (HDL), serum uric acid (SUA), estimated glomerular filtration rate (eGFR) and urinary albumin-to-creatinine ratio (UACR)). The following variables were also considered: body mass index (BMI), medical history including hypertension, diabetes mellitus, cardiovascular disease (CVD), and chronic kidney disease (CKD), as well as smoking and drinking habits. Adults who participated in NHANES between 2007 and 2016 were included in this study (48,711 individuals). Specific inclusion and exclusion criteria are listed below:

Inclusion criteria: 1. age ≥ 18 years. 2. complete data on α-Klotho levels were provided. 3. provide complete survival data.

Exclusion criteria:1. participants lacking α-Klotho data (34,947). 2. participants lacking survival data (16 individuals). 3. individuals who did not meet the age requirement.

Ultimately, a total of 13,748 participants met the inclusion criteria and were included in the analysis.

### Measurement of α-Klotho

Frozen serum samples were stored at -80°C for a period spanning from 2007 to 2016. α-Klotho levels were assayed by the NHANES team between 2019 and 2020. The specific steps are as follows:

1. Reagents and antibodies: commercial enzyme-linked immunosorbent assay (ELISA) kits manufactured by IBL International (Japan) were used for the assay.
2. Detection steps: Serum samples were divided into two parts and measured separately. Two independent measurements were made for each sample and the average value was taken as the final result. If the difference between the two measurements exceeds 10%, the assay is repeated. The value of the quality control sample needs to be within two standard deviations of the specified value, and if it does not meet the requirements, the same re-measurement is required.
3. Sensitivity and reference range: the sensitivity of the ELISA kit was 4.33 pg/mL. a reference range was established by 114 healthy donor samples, ranging from 285.8 to 1,638.6 pg/mL, with a mean value of 698.0 pg/mL (14).
4. Data processing: log10 transformation was performed in this study in order to make the data for conform to normal distribution.

### Outcome variables

The NHANES dataset was matched to the National Death Index records to determine the survival of the participants. International Classification of Diseases-Tenth Revision was used to determine the cause of death. Follow-up for each participant began at the time of the NHANES baseline interview and continued until death or the last follow-up visit, which occurred on 31 December 2019.

### Covariates

The NHANES database provides a comprehensive range of data from 2007 to 2016, encompassing questionnaires, physical measurements, laboratory measurements, and dietary information. In the context of this study, demographic data, including gender, age, race, educational attainment, and economic status (as indicated by the poverty income ratio (PIR)), was gathered through the administration of questionnaires. Furthermore, blood biomarkers, including FBG, HBA1C, SUA, TC, TG, HDL, and LDL, were quantified. Urine markers, specifically urinary creatinine and urinary albumin, were collected for the purpose of facilitating the calculation of the urinary albumin to creatinine ratio (UACR). Moreover, the estimated glomerular filtration rate (eGFR) was calculated using the CKD-EPI formula (15). Body measurements, including weight and height, were employed to ascertain the body mass index (BMI).

The past medical history was determined through a combination of physical examinations, self-reporting, and an analysis of the prescription medications that the participants were taking. In summary, hypertension was defined as three blood pressure measurements (systolic ≥ 140 mmHg or diastolic ≥ 90 mmHg) or a diagnosis of hypertension by a medical professional or the use of antihypertensive medication. A diagnosis of diabetes mellitus was defined as having been informed by a medical practitioner that the individual in question has diabetes mellitus or is taking medication to control their blood glucose levels. The presence of CVD was determined based on the participant’s history of congestive heart failure, coronary heart disease, or angina/angina pectoris. CKD was defined as an eGFR of less than 60 ml/min/1.73 m² and a UACR of 30 mg/g or greater (16).

The questionnaire was used to determine the definitions of smoking and alcohol consumption. Individuals who smoked more than 100 cigarettes during their lifetime were classified as smokers. On the other hand, those who consumed more than 12 drinks within a year were classified as alcohol drinkers.

### Statistical analysis

Dummy variables were set to indicate missing covariate values to avoid losing sample size. Continuous variables were tested for normality using the Lilliefors test. The continuous variables in this study (including age, PIR, α-Klotho, FBG, HBA1C, SUA, TC, TG, HDL, LDL, UACR, eGFR, and BMI) were not normally distributed. Therefore, these variables were expressed using medians (interquartile ranges). Comparisons between groups were made using the Kruskal-Wallis rank sum test. Categorical variables were expressed as percentages (%), and comparisons between groups were made using the Fisher exact probability test. Kaplan-Meier (KM) curves were used to demonstrate participant survival between log α-Klotho four groups, and survival was compared between groups using Cox regression analysis.

Three models were fitted for this study, with Model I adjusted for age (continuous), gender (male/female), race (Non-Hispanic white, Non-Hispanic black, Mexican American, and Other), education (junior high school education or below, high school education and college education or above) and PIR (continuous) were adjusted. Model II was further adjusted for FBG, HBA1C, SUA, TG, TC, HDL, LDL, UACR, eGFR, BMI, smoking and drinking habits, and disease history (hypertension, diabetes, CVD, and CKD). Model III was further adjusted for a set of dummy variables. The above adjustments for potential confounders were made based on existing literature (6, 9–12, 17) and clinical observations.

To evaluate the nonlinear association between log α-Klotho and the risk of death, we employed a restricted cubic spline (RCS) Cox proportional hazards regression model. Initially, a Cox model incorporating RCS was fitted to capture potential nonlinear relationships. Nonlinearity was assessed by comparing the RCS model to a traditional linear Cox model using a likelihood ratio test, with a significant p-value (p < 0.05) indicating deviation from linearity. Upon confirming nonlinearity, a threshold effects analysis was conducted to identify the optimal inflection point of log α-Klotho that best delineates the risk of death. Furthermore, three models were constructed to account for potential confounders: Model I adjusted for demographic variables, Model II further incorporated biochemical indicators and lifestyle factors, and Model III included additional dummy variables. To ensure robustness and reproducibility, sensitivity analyses were performed, and the consistency of the inflection points across different models was verified.

We conducted multiple sensitivity analyses to ensure the accuracy and consistency of our findings. Firstly, we excluded participants who had passed away within two years of follow-up to prevent any potential bias related to reverse causality. Secondly, we created subgroups based on different follow-up periods. Lastly, we stratified the analyses based on participants’ demographic characteristics such as age and sex, as well as their disease history, including hypertension, diabetes, CVD, and CKD.

The software tools used for all the above analyses were R (version 4.3.1) and Empower Stats (version 4.2). A two-sided P < 0.05 was considered statistically significant.

## Results

### Demographic and clinical baseline characteristics of the study population

The study included a total of 13,748 participants, of whom 1,569 died. The participants were followed for 5.33 years, with a range of 2.83 to 7.83 years. Those who died were mainly older and more likely to be male. They also had lower levels of α-Klotho and higher levels of FBG, HBA1C, SUA, TG, LDL and UACR compared to their living counterparts. In addition, the deceased participants had higher prevalence probabilities of hypertension, diabetes, CVD and CKD, and were more likely to smoke tobacco and consume alcohol (**Table 1**).

**Table 1:** Baseline characteristics of 2007-2016 NHANES enrolled study subjects grouped according to survival status.

Mortality	No	Yes	P-value
N	12179	1569	
Age (years)	56.00 (48.00-65.00)	68.00 (60.00-74.00)	<0.001
Sex [N (%)]			<0.001
Female	6445 (52.92%)	646 (41.17%)	
Male	5734 (47.08%)	923 (58.83%)	
Ethnic [N (%)]			<0.001
Non-Hispanic white	2025 (16.63%)	160 (10.20%)	
Non-Hispanic black	2372 (19.48%)	354 (22.56%)	
Mexican American	5068 (41.61%)	849 (54.11%)	
Other	2714 (22.28%)	206 (13.13%)	
Education [N (%)]			<0.001
Junior high school education or below	3301 (27.12%)	586 (37.42%)	
High school education	2668 (21.92%)	379 (24.20%)	
College education or above	6205 (50.97%)	601 (38.38%)	
PIR (%)	2.31 (1.16-4.54)	1.52 (0.98-2.88)	<0.001
α-Klotho (pg/mL)	806.70 (659.80-997.60)	761.50(602.60-957.00)	<0.001
Log α-Klotho (pg/mL)	2.91 (2.82-3.00)	2.88 (2.78-2.98)	<0.001
FBG (mmol/L)	5.72 (5.27-6.38)	6.05 (5.44-7.22)	<0.001
HBA1C (%)	5.70 (5.40-6.00)	5.80 (5.40-6.50)	<0.001
SUA (μmol/L)	321.20(267.70-380.70)	345.00(285.50-416.40)	<0.001
TG(mmol/L)	1.23 (0.86-1.79)	1.32 (0.93-1.91)	0.040
TC(mmol/L)	5.12 (4.42-5.82)	4.78 (4.06-5.64)	<0.001
HDL(mmol/L)	1.32 (1.09-1.60)	1.24 (1.01-1.55)	<0.001
LDL(mmol/L)	3.00 (2.43-3.65)	2.71 (2.02-3.44)	<0.001
UACR (mg/g)	7.31 (4.81-13.79)	13.11 (6.67-45.00)	<0.001
eGFR(mL/min/1.73m^2^)	90.50 (76.18-102.18)	76.09 (58.86-91.64)	<0.001
BMI (kg/m^2^)	28.70 (25.20-33.10)	28.80 (24.83-33.45)	0.630
Follow-up time (years)	8.00 (5.50-10.46)	5.33 (2.83-7.83)	<0.001
Smoking [N (%)]			<0.001
No	6533 (53.67%)	532 (33.93%)	
Yes	5640 (46.33%)	1036 (66.07%)	
Drinking [N (%)]			0.421
No	1664 (14.75%)	204 (13.96%)	
Yes	9614 (85.25%)	1257 (86.04%)	
Hypertension [N (%)]			<0.001
No	5952 (48.88%)	409 (26.07%)	
Yes	6226 (51.12%)	1160 (73.93%)	
Diabetes mellitus[N (%)]			<0.001
No	9380 (77.07%)	930 (59.31%)	
Yes	2791 (22.93%)	638 (40.69%)	
CVD[N (%)]			<0.001
No	10822 (88.87%)	1019 (64.95%)	
Yes	1355 (11.13%)	550 (35.05%)	
CKD[N (%)]			<0.001
No	10016 (82.70%)	826 (54.02%)	
Yes	2095 (17.30%)	703 (45.98%)	
Cause of death[N (%)]			
Accidents (unintentional injuries)	-	32 (2.04%)	
All other causes (residual)	-	419 (26.70%)	
Alzheimer’s disease	-	31 (1.98%)	
Cerebrovascular diseases	-	92 (5.86%)	
Chronic lower respiratory diseases	-	78 (4.97%)	
Diabetes mellitus	-	64 (4.08%)	
Diseases of heart	-	353 (22.50%)	
Influenza and pneumonia	-	34 (2.17%)	
Malignant neoplasms	-	443 (28.23%)	
Nephritis, nephrotic syndrome and nephrosis	-	23 (1.47%)	

Continuous variables are expressed using medians (Q1-Q3) and categorical variables are expressed using percentages.

PIR, poverty income ratio; FBG, fasting blood glucose; HbA1C, hemoglobin A1c; SUA, serum uric acid; TG, triglyceride; TC, total cholesterol; HDL, high-density lipoprotein; LDL, low-density lipoprotein; UACR, urinary albumin-to-creatinine ratio; eGFR, estimated glomerular filtration rate; BMI, body mass index; CVD, cardiovascular disease; CKD, chronic kidney disease.

Examination of the log α-Klotho quartile groups showed that individuals in quartile 1 (2.18-2.81 pg/mL) had an older age and higher levels of FBG, HBA1C, SUA, TG, UACR and UACR. There was also an increased incidence of hypertension, diabetes, CVD and CKD, as well as higher proportions of smoking and alcohol consumption (**Table 2**).

**Table 2:** Baseline characteristics of study participants included in NHANES 2007-2016 grouped according to α-Klotho quartiles.

Log α-Klotho quartile (pg/mL)	Quartile 1 2.18-2.81	Quartile 2 2.82-2.90	Quartile 3 2.91-2.99	Quartile 4 3.0-3.70	P-value
N	3437	3436	3438	3437	
Age	60.00 (50.00-68.00)	58.00 (49.00-66.00)	57.00 (48.00-66.00)	56.00 (47.00-64.00)	<0.001
Sex					<0.001
Female	1664 (48.41%)	1659 (48.28%)	1811 (52.68%)	1957 (56.94%)	
Male	1773 (51.59%)	1777 (51.72%)	1627 (47.32%)	1480 (43.06%)	
Ethnic [N (%)]					<0.001
Non-Hispanic white	556 (16.18%)	538 (15.66%)	560 (16.29%)	531 (15.45%)	
Non-Hispanic black	698 (20.31%)	568 (16.53%)	585 (17.02%)	875 (25.46%)	
Mexican American	1554 (45.21%)	1590 (46.27%)	1503 (43.72%)	1270 (36.95%)	
Other	629 (18.30%)	740 (21.54%)	790 (22.98%)	761 (22.14%)	
Education [N (%)]					0.001
Junior high school education or below	1009 (29.39%)	967 (28.15%)	951 (27.69%)	960 (27.93%)	
High school education	827 (24.09%)	745 (21.69%)	771 (22.45%)	704 (20.48%)	
College education or above	1597 (46.52%)	1723 (50.16%)	1713 (49.87%)	1773 (51.59%)	
PIR (%)	2.11 (1.11-4.19)	2.24 (1.16-4.43)	2.27 (1.16-4.46)	2.22 (1.13-4.37)	0.048
FBG (mmol/L)	5.77 (5.27-6.38)	5.72 (5.33-6.44)	5.72 (5.33-6.38)	5.77 (5.33-6.61)	<0.001
HBA1C (%)	5.70 (5.40-6.10)	5.70 (5.40-6.00)	5.60 (5.40-6.00)	5.70 (5.40-6.10)	<0.001
SUA (μmol/L)	333.10 (279.60-398.50)	327.10 (273.60-386.60)	321.20 (267.70-374.70)	303.30 (249.80-362.80)	<0.001
TG (mmol/L)	1.30 (0.91-1.91)	1.28 (0.91-1.86)	1.24 (0.87-1.78)	1.15 (0.81-1.69)	<0.001
TC (mmol/L)	5.07 (4.32-5.87)	5.09 (4.40-5.79)	5.07 (4.42-5.79)	5.07 (4.40-5.77)	0.920
HDL (mmol/L)	1.29 (1.06-1.60)	1.29 (1.06-1.58)	1.29 (1.06-1.60)	1.32 (1.09-1.63)	0.011
LDL (mmol/L)	2.92 (2.30-3.60)	3.00 (2.43-3.64)	3.00 (2.43-3.65)	2.97 (2.40-3.60)	0.031
UACR (mg/g)	7.89 (4.90-16.94)	7.67 (5.00-15.19)	7.40 (4.77-14.27)	7.74 (5.08-15.26)	<0.001
eGFR(mL/min/1.73m^2^)	84.96 (68.27-98.65)	88.65 (73.37-100.48)	90.01 (75.92-101.82)	92.29 (79.08-104.09)	<0.001
BMI (kg/m^2^)	28.90 (25.50-33.20)	28.80 (25.22-33.18)	28.60 (25.10-33.00)	28.60 (24.73-33.20)	0.499
Follow-up time (years)	7.42 (4.67-10.25)	7.67 (5.17-10.33)	7.83 (5.42-10.33)	7.92 (5.42-10.25)	<0.001
Smoking [N (%)]					<0.001
No	1572 (45.79%)	1698 (49.43%)	1829 (53.22%)	1966 (57.22%)	
Yes	1861 (54.21%)	1737 (50.57%)	1608 (46.78%)	1470 (42.78%)	
Drinking [N (%)]					<0.001
No	399 (12.40%)	469 (14.72%)	454 (14.31%)	546 (17.27%)	
Now	2820 (87.60%)	2718 (85.28%)	2718 (85.69%)	2615 (82.73%)	
Hypertension [N (%)]					<0.001
No	1433 (41.69%)	1605 (46.71%)	1669 (48.56%)	1654 (48.12%)	
Yes	2004 (58.31%)	1831 (53.29%)	1768 (51.44%)	1783 (51.88%)	
Diabetes mellitus[N (%)]					0.016
No	2536 (73.83%)	2621 (76.32%)	2617 (76.12%)	2536 (73.89%)	
Yes	899 (26.17%)	813 (23.68%)	821 (23.88%)	896 (26.11%)	
CVD [N (%)]					<0.001
No	2840 (82.65%)	2965 (86.29%)	2994 (87.09%)	3042 (88.53%)	
Yes	596 (17.35%)	471 (13.71%)	444 (12.91%)	394 (11.47%)	
CKD [N (%)]					<0.001
No	2494 (73.18%)	2711 (79.43%)	2826 (82.95%)	2811 (82.39%)	
Yes	914 (26.82%)	702 (20.57%)	581 (17.05%)	601 (17.61%)	
Mortality [N (%)]					<0.001
No	2932 (85.31%)	3071 (89.38%)	3090 (89.88%)	3086 (89.79%)	
Yes	505 (14.69%)	365 (10.62%)	348 (10.12%)	351 (10.21%)	
Cause of death [N (%)]					0.482
Accidents (unintentional injuries)	8 (1.58%)	11 (3.01%)	8 (2.30%)	5 (1.42%)	
All other causes (residual)	133 (26.34%)	91 (24.93%)	92 (26.44%)	103 (29.34%)	
Alzheimer’s disease	7 (1.39%)	10 (2.74%)	4 (1.15%)	10 (2.85%)	
Cerebrovascular diseases	33 (6.53%)	15 (4.11%)	25 (7.18%)	19 (5.41%)	
Chronic lower respiratory diseases	25 (4.95%)	23 (6.30%)	17 (4.89%)	13 (3.70%)	
Diabetes mellitus	17 (3.37%)	15 (4.11%)	13 (3.74%)	19 (5.41%)	
Diseases of heart	121 (23.96%)	91 (24.93%)	75 (21.55%)	66 (18.80%)	
Influenza and pneumonia	12 (2.38%)	5 (1.37%)	7 (2.01%)	10 (2.85%)	
Malignant neoplasms	139 (27.52%)	101 (27.67%)	104 (29.89%)	99 (28.21%)	
Nephritis, nephrotic syndrome, and nephrosis	10 (1.98%)	3 (0.82%)	3 (0.86%)	7 (1.99%)	

Continuous variables are expressed using medians (Q1-Q3) and categorical variables are expressed using percentages.

PIR, poverty income ratio; FBG, fasting blood glucose; HbA1C, hemoglobin A1c; SUA, serum uric acid; TG, triglyceride; TC, total cholesterol; HDL, high-density lipoprotein; LDL, low-density lipoprotein; UACR, urinary albumin-to-creatinine ratio; eGFR, estimated glomerular filtration rate; BMI, body mass index; CVD, cardiovascular disease; CKD, chronic kidney disease.

### Association of log α-Klotho with all-cause mortality in NHANES 2006-2017

**Figure 1:**
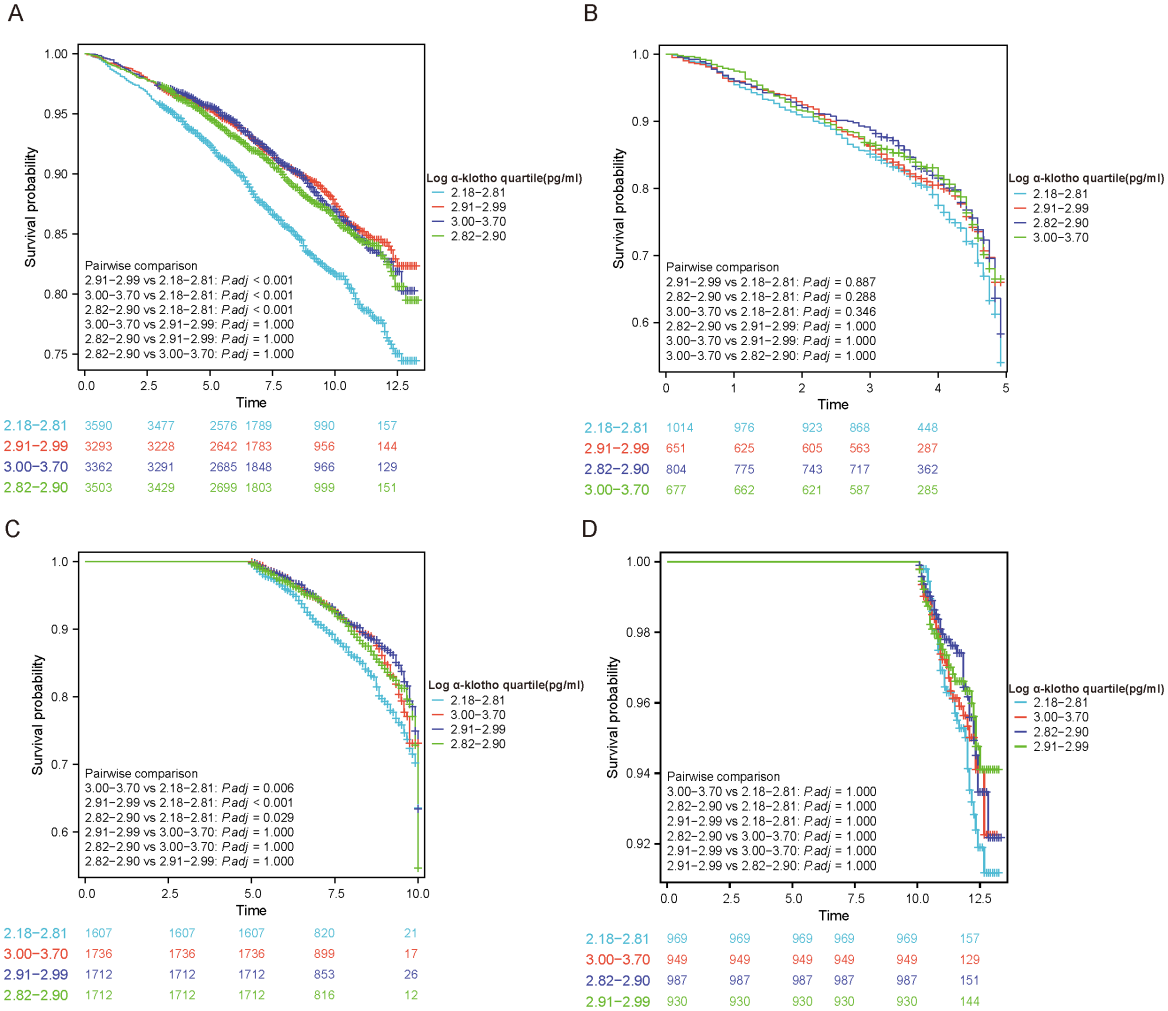
KM curves to analyze outcomes of included study subjects. **(A)** Analysis based on α-Klotho quartiles; **(B-D)** Analysis based on duration of follow-up (<5, 5-10 and >10 years).

KM curves were utilized to illustrate the correlation between pairwise comparisons of log α-Klotho and all-cause mortality. It was observed that individuals falling within the Quartile 1 range of log α-Klotho exhibited the lowest survival rate (P<0.05) (**Figure 1A**). Similar outcomes were observed in participants with a follow-up period of 5-10 years (**Figure 1C**), indicating a potential association between lower α-Klotho levels and decreased mortality risk. However, similar results were not observed in participants with follow-up periods of less than 5 years and greater than 10 years (**Figures 1B**, **D**). Subsequently, univariate Cox regression analysis was conducted to examine the variables associated with participants’ susceptibility to all-cause mortality (**Supplementary Table 2**). Our study shows a weak association between log α-Klotho and all-cause mortality.

To further investigate the independent role of log α-Klotho in the risk of all-cause mortality, multifactor Cox regression analysis was conducted, as presented in **Supplementary Table 3**. Controlling for various confounders and dummy variables (**Supplementary Table 1**), the risk of all-cause mortality was significantly reduced by 44% for each 1 pg/mL increase in log α-Klotho. Additionally, the investigation involved the examination of the correlation between log α-Klotho and the risk of mortality by dividing log α-Klotho into quartiles. The results revealed that only in quartile 1 (2.18-2.81 pg/mL) was log α-Klotho weakly linked to a reduced risk of all-cause mortality. Conversely, log α-Klotho in quartile 2 (2.82-2.90 pg/mL), quartile 3 (2.91-2.99 pg/mL), and quartile 4 (3.0-3.70 pg/mL) did not exhibit any association with a decreased risk of all-cause mortality. Furthermore, we conducted a comprehensive examination of the correlation between log α-Klotho and the probability of mortality over different follow-up periods. Our findings indicate that log α-Klotho was not significantly associated with all-cause mortality within follow-up periods of <5 years, 5-10 years, and >10 years.

### Log α-Klotho showed U-shaped non-linear relationship with risk of all-cause mortality

**Figure 2:**
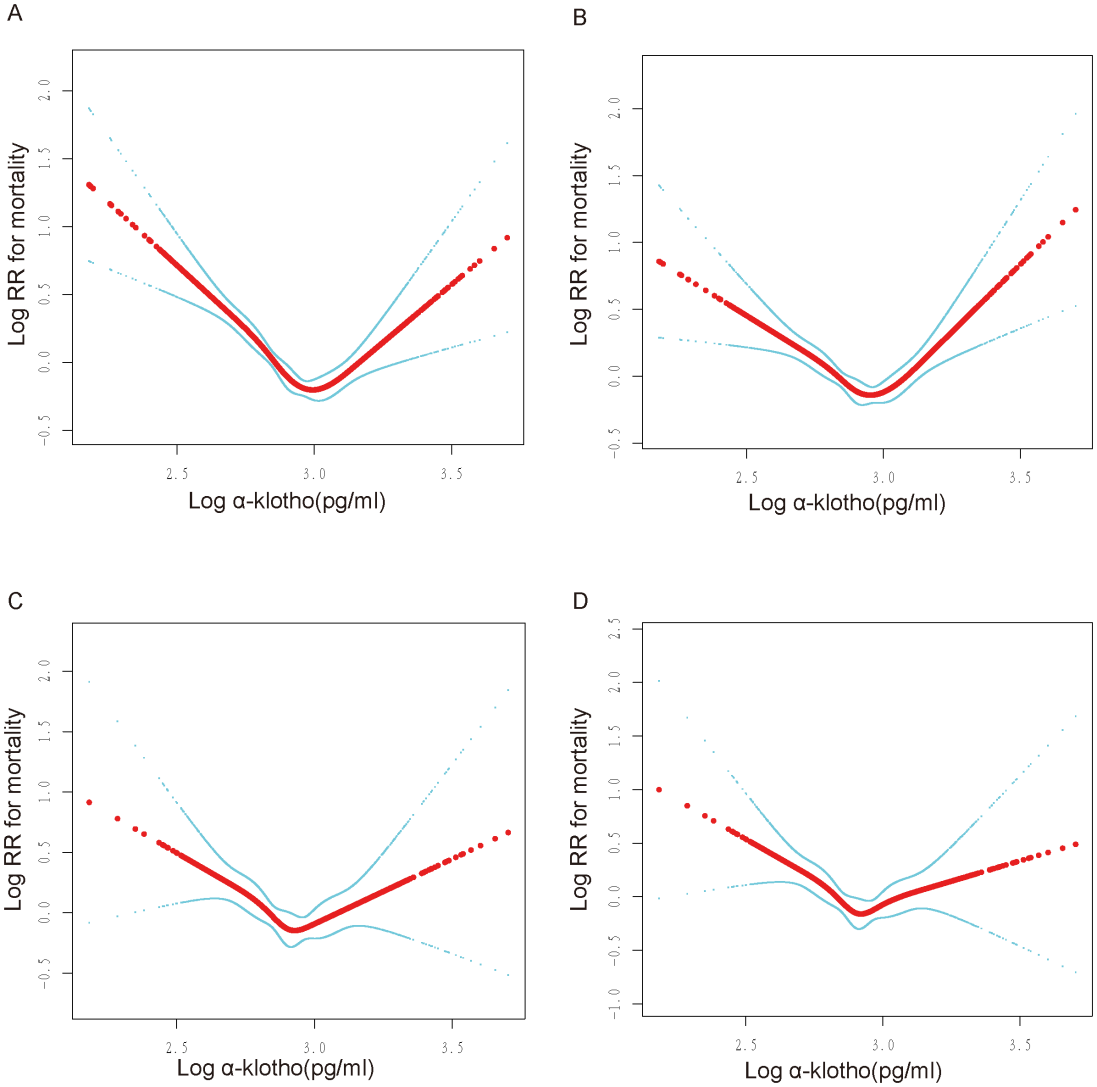
U-shaped non-linear relationship between log α-Klotho and mortality. RR: relative risk. **(A)** No variables were adjusted. **(B)** Adjusted for age, sex, ethnic, poverty income ratio, education. **(C)** Adjusted for Model 1+fasting blood glucose, hemoglobin A1c, serum uric acid, triglyceride, total cholesterol, high-density lipoprotein, low-density lipoprotein, urinary albumin-to-creatinine ratio, estimated glomerular filtration rate, body mass index, smoking, drinking, hypertension, diabetes, cardiovascular disease, chronic kidney disease. **(D)** Adjusted for Model 2+poverty income ratio dummy variable, fasting blood glucose dummy variable, hemoglobin A1c dummy variable, triglyceride dummy variable, high-density lipoprotein dummy variable, low-density lipoprotein dummy variable, urinary albumin-to-creatinine ratio dummy variable, estimated glomerular filtration rate dummy variable, body mass index dummy variable, hypertension dummy variable, diabetes mellitus dummy variable, cardiovascular disease dummy variable, chronic kidney disease dummy variable, smoking dummy variable, drinking dummy variable.

The present study examined the association between log α-Klotho and the risk of all-cause mortality, taking into account various confounding factors. Our findings revealed a non-linear relationship, characterized by a U-shaped pattern, between log α-Klotho and the risk of all-cause mortality, as depicted in **Figures 2A–D**. Furthermore, through threshold effect analysis, we identified the optimal inflection point, as presented in **Table 3**. Specifically, when log α-Klotho levels were below 2.89 pg/mL, the risk of all-cause mortality decreased by 86%. Conversely, log α-Klotho levels exceeding 2.89 pg/mL were associated with a 2.15-fold increase in the risk of all-cause mortality.

**Table 3:** Two-piecewise Cox proportional risk regression analysis the effect of all-cause mortality on in NHANES 2007-2016.

Outcome:	All-cause mortality		
	HR (95%CI)	P Value	P non-linear value (P for log-likelihood ratio test)
Crude			
Log α-Klotho < 3 pg/mL	0.13 (0.09, 0.21)	<0.0001	<0.001
Log α-Klotho > 3 pg/mL	5.56 (2.35, 13.14)	<0.0001	
Model 1			
Log α-Klotho< 2.99 pg/mL	0.27 (0.17, 0.42)	<0.0001	<0.001
Log α-Klotho < 2.99 pg/mL	8.02 (3.41, 18.88)	<0.0001	
Model 2			
Log α-Klotho < 2.96 pg/mL	0.23 (0.10, 0.50)	0.0003	0.002
Log α-Klotho < 2.96 pg/mL	3.55 (1.04, 12.20)	0.0439	
Model 3			
Log α-Klotho < 2.89 pg/mL	0.14 (0.05, 0.38)	0.0001	0.002
Log α-Klotho < 2.89 pg/mL	2.15 (0.81, 5.72)	0.1252	
Follow-up time < 5 years			
Log α-Klotho < 2.86 pg/mL	0.04 (0.01, 0.17)	<0.0001	<0.001
Log α-Klotho < 2.86 pg/mL	4.76 (1.44, 15.77)	0.0106	
Follow-up time 5-10 years			
Log α-Klotho < 3.16 pg/mL	0.34 (0.14, 0.85)	0.0207	0.002
Log α-Klotho < 3.16 pg/mL	1427.72 (21.73, 93818.47)	0.0007	
Follow-up time > 5 years			
Log α-Klotho < 2.98 pg/mL	0.20 (0.01, 5.86)	0.3532	0.093
Log α-Klotho < 2.98 pg/mL	89.79 (0.80, 10130.14)	0.0621	

Model 1: Adjusted for age, sex, ethnic, poverty income ratio, education.

Model 2: Adjusted for Model 1+fasting blood glucose, hemoglobin A1c, serum uric acid, triglyceride, total cholesterol, high-density lipoprotein, low-density lipoprotein, urinary albumin-to-creatinine ratio, estimated glomerular filtration rate, body mass index, smoking, drinking, hypertension, diabetes, cardiovascular disease, chronic kidney disease.

Model 3: Adjusted for Model 2+poverty income ratio dummy variable, fasting blood glucose dummy variable, hemoglobin A1c dummy variable, triglyceride dummy variable, high-density lipoprotein dummy variable, low-density lipoprotein dummy variable, urinary albumin-to-creatinine ratio dummy variable, estimated glomerular filtration rate dummy variable, body mass index dummy variable, hypertension dummy variable, diabetes mellitus dummy variable, cardiovascular disease dummy variable, chronic kidney disease dummy variable, smoking dummy variable, drinking dummy variable.

**Figure 3:**
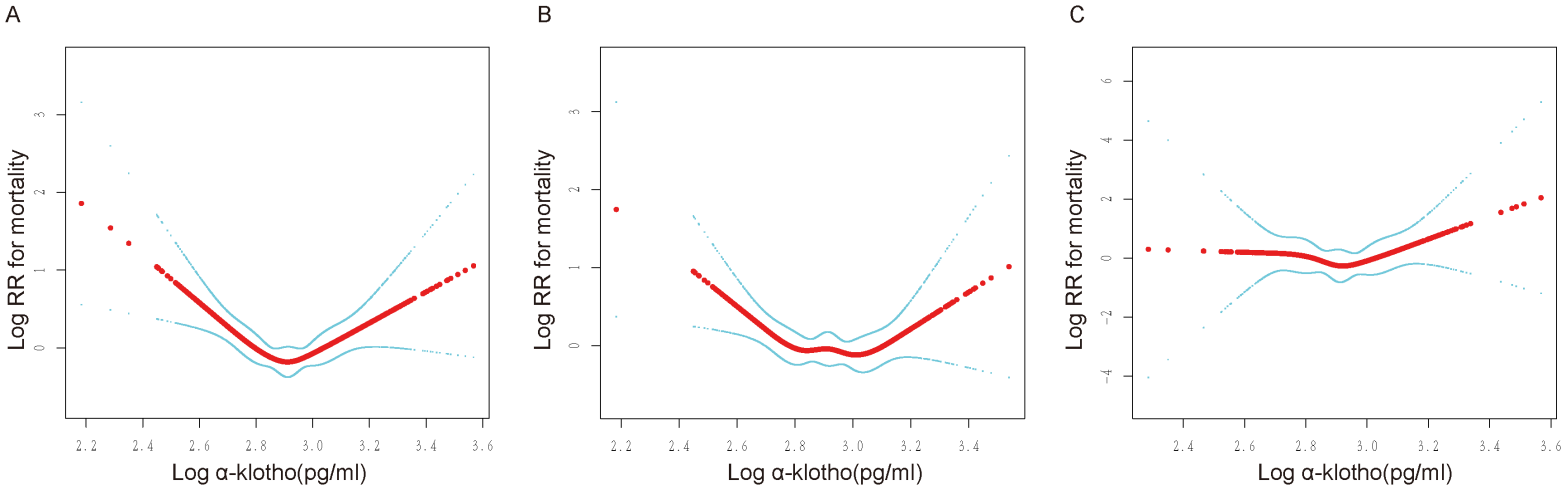
Non-linear relationship between log α-Klotho and mortality at different follow-up durations. RR: relative risk. **(A)** Association of log α-Klotho with mortality in less than 5 years, **(B)** Association of log α-Klotho with mortality over 5-10 years, **(C)** Association of log α-Klotho with mortality in >10 years. Adjusted for age, sex, ethnic, poverty income ratio, education, fasting blood glucose, hemoglobin A1c, serum uric acid, triglyceride, total cholesterol, high-density lipoprotein, low-density lipoprotein, urinary albumin-to-creatinine ratio, estimated glomerular filtration rate, body mass index, smoking, drinking, hypertension, diabetes, cardiovascular disease, chronic kidney disease, poverty income ratio dummy variable, fasting blood glucose dummy variable, hemoglobin A1c dummy variable, triglyceride dummy variable, high-density lipoprotein dummy variable, low-density lipoprotein dummy variable, urinary albumin-to-creatinine ratio dummy variable, estimated glomerular filtration rate dummy variable, body mass index dummy variable, hypertension dummy variable, diabetes mellitus dummy variable, cardiovascular disease dummy variable, chronic kidney disease dummy variable, smoking dummy variable, drinking dummy variable.

In addition, a sensitivity analysis was conducted to exclude participants who died within two years of follow-up to limit the effect of reverse causality. Notably, the U-shaped association between log α-Klotho and all-cause mortality remained consistent, as depicted in **Supplementary Figure 1**. Additionally, participants were categorized based on the duration of follow-up, revealing that the U-shaped relationship between log α-Klotho and all-cause mortality remained unchanged across follow-up periods of less than five years, 5-10 years, and exceeding ten years, as illustrated in **Figure 3**. After stratifying the data by age, sex, and medical history, including hypertension, diabetes, CVD, and CKD, a U-shaped correlation between log α-Klotho and mortality risk was still observed (**Supplementary Figures 2**, **3**). Nevertheless, the statistical significance of this U-shaped relationship was not consistently observed in certain subgroups, primarily due to the constraints imposed by the limited sample size. Moreover, **Supplementary Table 4** presents the inflection points for the various stratifications.

## Discussion

The aim of our study was to examine the association between log α-Klotho levels and the likelihood of mortality. In a retrospective analysis, we observed a modest association between log α-Klotho and a reduced risk of mortality, particularly in individuals in the first quartile. In addition, we examined the dose-response relationship between log α-Klotho levels and mortality risk and found a U-shaped association while controlling for various confounders. We also identified the optimal inflection point for log α-Klotho at 2.89 pg/mL. Finally, we demonstrated the stability of our findings in various stratification and sensitivity analyses.

There is currently no precise value for the normal range of α-Klotho. Yamazaki Y (18) measured circulating serum α-Klotho levels by ELISA. His (18) study found that normal serum α-Klotho levels in adults ranged from 239-1266 pg/mL (mean ± standard deviation 562 ± 146 pg/mL). Later, Drew-DA (19) also used an ELISA to measure circulating serum α-Klotho levels. His (19) study found a median α-Klotho level of 631 pg/mL (25th-75th percentile 477-817 pg/mL). However, the specificity of the ELISA assay may be lower than that of immunoprecipitant immunoblotting assays (20). The reference range for α-Klotho in the NHANES database is 285.8-1638.6 pg/mL, which is in general agreement with the results of the above study. In addition, there is still a lack of a robust and credible assay for circulating α-Klotho, and scientists have expressed concern about the accuracy of commercially available kits (21). The advent of new antibodies (22), branched polypeptides (23) and other assays is expected to provide more accurate results.

Klotho is an anti-aging protein, and transgenic mice that overexpress α-Klotho have a 30% longer lifespan (1). Klotho has organ-protective effects. Klotho deficiency makes the kidneys more susceptible to acute injury, delays renal regeneration, and promotes renal fibrosis. In addition to direct renal effects, Klotho deficiency triggers and exacerbates disorders of mineral metabolism, secondary hyperparathyroidism, vascular calcification, and cardiac hypertrophy and fibrosis (24). Moreover, Klotho expression is significantly decreased in malignant tumors, and low Klotho levels are an independent poor prognostic factor for cancer-specific and progression-free survival (25). Klotho deficiency has been associated with several neurological disorders, including multiple sclerosis, Alzheimer’s disease, amyotrophic lateral sclerosis, and Parkinson’s disease (26). It has been found that Alzheimer’s disease patients have lower Klotho concentrations in their cerebrospinal fluid than healthy individuals (27). Klotho-overexpressing mice have 1.76 times higher axon density than wild-type mice, which suggests a substantial beneficial role of Klotho proteins in myelin sheath regeneration (28). Klotho also enhances cognitive performance in mammals (29). In summary, low levels of Klotho are not beneficial for physical health. In fact, in our study, participants with low levels of α-Klotho had a higher prevalence of hypertension, diabetes, CVD and CKD, as well as mortality.

Several previous studies have examined the relationship between Klotho and mortality risk. However, the results of these studies are contradictory. Plasma Klotho is an independent predictor of all-cause mortality in the elderly (9). During hemodialysis, Klotho < 280 pg/mL will significantly increase the incidence of combined cardiovascular time and cardiovascular death (30). In addition, low concentrations of Klotho predicted CVD death in non-calcified and mildly calcified hemodialysis patients. In CKD patients, the low Klotho concentration group had a reduced probability of survival and an increased risk of all-cause mortality than the high Klotho concentration group (31). However, it has also been noted that serum α-Klotho is not associated with a higher risk of mortality (11, 12). A study by Brandenburg Vincent M et al. also noted that Klotho does not increase the predictive power of cardiovascular and mortality risk assessment in patients with normal renal function (32). In the present study, we revealed a U-shaped association between α-Klotho levels and mortality risk in the general population. In addition, consistent associations between α-Klotho and mortality risk were observed in different subsets of the population regardless of sex, age, and presence of hypertension, diabetes, CKD, or CVD. This finding may shed light on the inconsistent findings of previous studies regarding the relationship between Klotho and mortality risk.

Several studies have explained the Klotho association with mortality risk. More than ten single nucleotide polymorphisms (SNPs) have been identified in the human Klotho gene, and numerous studies have been conducted to assess the association between allelic variation in the Klotho gene and the etiology of aging-related diseases (33–35). Gang Jee Ko’s (36) study found that in the promoter region of Klotho, G395A, the A allele carrier status is a factor affecting the survival rate of hemodialysis patients, and the survival rate of the GA+AA group was lower than that of the GG group. A study by Serafi Cambray (37) also identified three SNPs in Klotho: rs562020 carrying the most common allele (G), a rare allele (C) at rs2283368, and a pureblood at rs2320762 rare allele (G), which could help in the prediction of non-cardiovascular mortality in CKD. In addition, the involvement of low concentrations of Klotho in the progression of various diseases also explains the relationship between Klotho and mortality.

Numerous scientists have worked to investigate the underlying mechanisms of how low Klotho levels increase the risk of death, and Stephanie S. Fischer (38) and colleagues have proposed that Klotho deficiency leads to severe disturbances in mineral and electrolyte metabolism, as well as lowered blood pressure, in mice. In addition, knockdown of the Klotho gene can promote neuronal cell death by regulating oxidative stress (39). Low Klotho levels have also been associated with accelerated aging and premature death by affecting the immune system (40). Despite the existence of a large body of basic research, there is no consensus in the scientific community on how low Klotho levels contribute to premature death.

Klotho overexpression has been demonstrated to attenuate the progression of various diseases. Specifically, it has been found to mitigate the physiologic compensatory hypertrophy of the kidney following nephrectomy and has shown efficacy in reducing disease progression (41). Additionally, Klotho overexpression has been observed to hinder cell proliferation and promote apoptosis in A549 cells, suggesting its potential as a tumor suppressor (42). Moreover, the inclusion of Klotho has been acknowledged as a potential therapeutic approach for conditions such as multiple sclerosis, Duchenne muscular dystrophy, cardiovascular disease, and osteoarthritis, and neuronal damage (43–47). Certain drugs have demonstrated the ability to enhance Klotho levels. For instance, Astragaloside IV has been found to elevate Klotho expression, ameliorate renal function, and mitigate podocyte apoptosis and injury in mice with diabetic nephropathy (48). Simvastatin alleviated cognitive dysfunction in rats and found a significant increase in klotho-positive cells in the hippocampus of simvastatin-treated rats (49). Rosiglitazone inhibits melanoma resistance by increasing serum levels of klotho and decreasing levels of Wnt5A in the blood and tumor microenvironment of mice (50). Consequently, the elevation of Klotho levels through external means presents a novel approach for alleviating symptoms and treating various diseases.

The U-shaped relationship between log α-Klotho levels and all-cause mortality observed in our study suggests that both low and high levels of α-Klotho may be detrimental to health, with intermediate levels being optimal. Several potential mechanisms may explain this phenomenon:

1. Regulation of Physiological Homeostasis:

Low levels of α-Klotho: α-Klotho is well-established for its antioxidant, anti-inflammatory, and anti-aging properties (1, 51). Reduced levels of α-Klotho may result in the loss of these protective effects, increasing the risk of various conditions such as cardiovascular disease, chronic kidney disease, and neurodegenerative disorders.

Excessive levels of α-Klotho: While moderate levels of α-Klotho are beneficial, excessive concentrations may disrupt mineral metabolism and calcium-phosphorus homeostasis, potentially leading to vascular calcification and other metabolic abnormalities (52). Furthermore, supraphysiological levels of α-Klotho may interfere with the feedback mechanisms of endocrine pathways, resulting in adverse effects.

2. Feedback Regulatory Mechanisms:

α-Klotho interacts with the FGF23/FGFR signaling pathway, which plays a key role in phosphate and vitamin D metabolism (52). Deviations from optimal α-Klotho levels may disrupt this pathway, leading to dysregulation of phosphate homeostasis. This imbalance can result in complications such as cardiovascular and renal dysfunction.

3. Genetic and Epigenetic Factors:

Genetic variations in the Klotho gene, such as single nucleotide polymorphisms (SNPs), can influence the expression and function of α-Klotho (33–36). These genetic differences may result in varying individual responses to α-Klotho levels, contributing to the observed U-shaped relationship. Additionally, epigenetic modifications that affect Klotho gene expression may further complicate this association.

4. Disease State Regulation:

α-Klotho levels undergo significant changes in various disease states, such as chronic kidney disease and heart failure (31, 32). These fluctuations may reflect either compensatory mechanisms or pathological processes, which could differentially affect mortality risk depending on the α-Klotho concentration.

5. External Factors and Interventions:

External factors, including pharmacological agents that influence α-Klotho levels (e.g., astragaloside IV, simvastatin, rosiglitazone) (48–50), may cause fluctuations in α-Klotho concentrations outside the optimal range. Such deviations may inadvertently increase the risk of adverse outcomes, including mortality, particularly at high α-Klotho levels.

6. Cellular and Molecular Effects:

At optimal levels, α-Klotho promotes cellular homeostasis, enhances cell survival, and inhibits apoptosis (42, 48). However, excessive α-Klotho levels may induce cellular stress, autophagy, or apoptosis, increasing the risk of tissue damage and adverse health outcomes.

These potential mechanisms collectively highlight the complex and context-dependent role of α-Klotho in human health, providing a theoretical framework for understanding its dual biological effects and implications for disease and mortality.

The present study offers several novel contributions. First, it leverages NHANES data from a nationally representative sample of 13,748 participants, significantly enhancing the external validity of the findings. Second, it is the first to confirm, within a general population, the U-shaped nonlinear relationship between serum α-Klotho levels and all-cause mortality, identifying a specific inflection point at 2.89 pg/mL. This finding sheds light on previously conflicting results and provides a clearer interpretation of the association. Furthermore, the robustness of this relationship was verified through multiple adjustments and sensitivity analyses, thereby reinforcing the study’s credibility. Lastly, this research delves into the bidirectional biological effects of α-Klotho at different concentrations, offering a valuable theoretical foundation for future basic research and clinical applications. Collectively, these contributions advance the understanding of α-Klotho in aging and disease while providing new perspectives for clinical risk assessment and the development of targeted interventions.

It is crucial to acknowledge certain limitations inherent in this observational study, which prevented us from establishing a causal relationship between α-Klotho and susceptibility to all-cause mortality. In addition, not all blood samples were collected on the same day and were not consistent. In addition, this blood sample was stored at -80°C without timely testing, which may pose a potential risk of protein degradation. Therefore, better study designs are needed in the future to demonstrate the relationship between α-Klotho and mortality risk. Furthermore, our exclusive reliance on a single baseline measurement of α-Klotho concentration has limited our ability to assess the influence of time-dependent fluctuations in α-Klotho on the risk of all-cause mortality. Next, the limited sample size for each specific cause of mortality has hindered our ability to examine the relationship between α-Klotho and mortality risk across different causes. It is crucial to acknowledge that residual and unidentified confounding factors may persist despite our efforts to minimize them. A substantial proportion of the sample had to be excluded due to the absence of data, consequently constraining the study’s capacity to accurately depict the US population.

## Conclusion

In a nationally representative U.S. population, we found a U-shaped association between circulating α-Klotho levels and risk of all-cause mortality, with a specific inflection point at 2.89 pg/mL. The results of different levels and sensitivity analyses supported this association. Future studies need to further explore the specific mechanisms of the effect of α-Klotho on mortality risk at different levels.

## Data Availability

The original contributions presented in the study are included in the article/**Supplementary Material**. Further inquiries can be directed to the corresponding author/s.
