# Supplementary material for: The inflection point: α-Klotho levels and the risk of all-cause mortality
Source: Front Endocrinol (Lausanne). 2025 Mar 11;16:1405003. doi: 10.3389/fendo.2025.1405003 (PMC11932894; doi:10.3389/fendo.2025.1405003)
Supplement: Supplementary file 1 [file DataSheet1.doc]

**Supplement figure 1 U-shaped nonlinear relationship between log α-Klotho and all-cause mortality(Exclusion of participants who death within 2 years)**

**
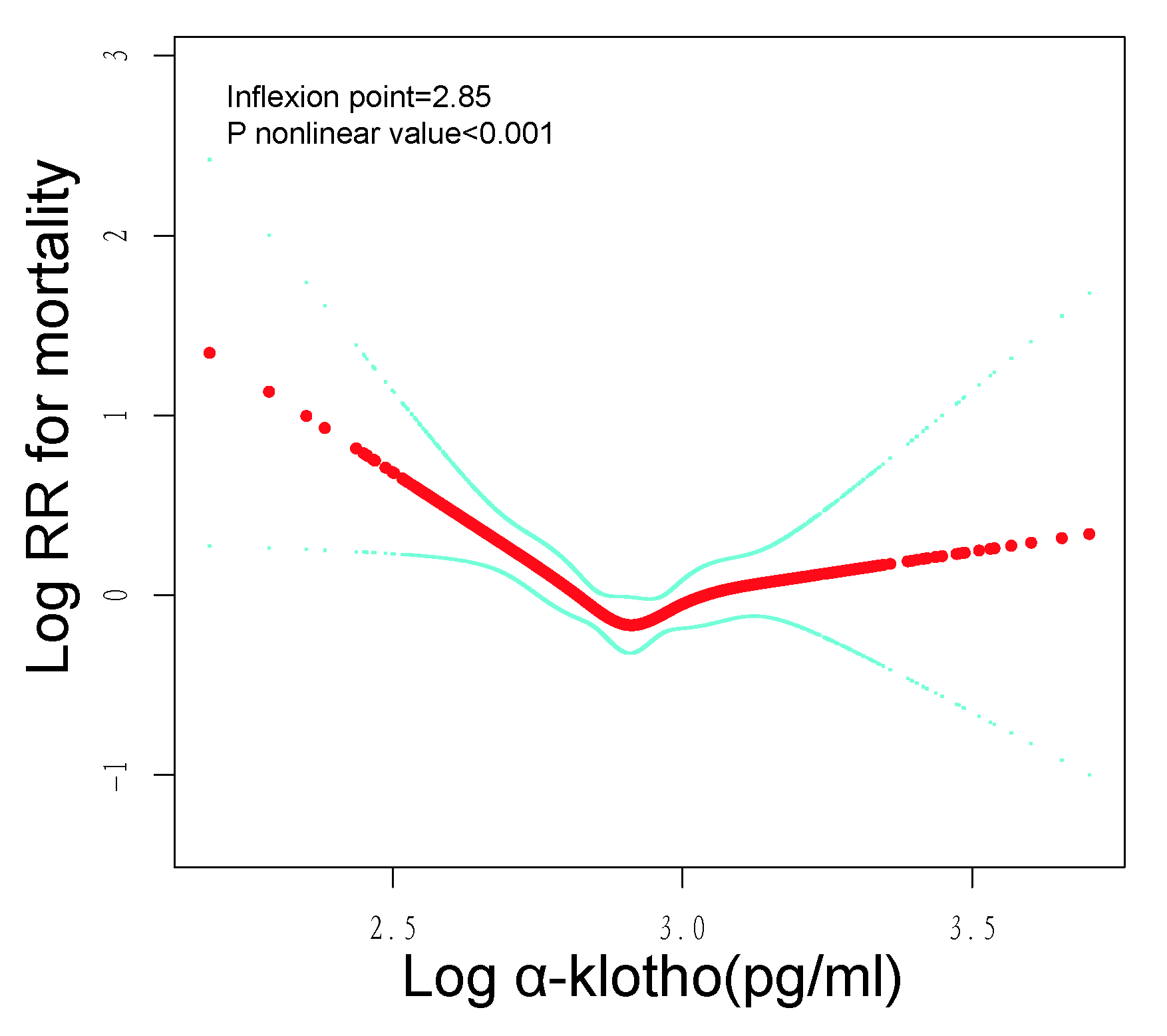
**

RR:relative risk.

Adjusted for age, sex, ethnic, poverty income ratio, education, fasting blood glucose, hemoglobin A1c, serum uric acid, triglyceride, total cholesterol, high-density lipoprotein, low-density lipoprotein, urinary albumin-to-creatinine ratio, estimated glomerular filtration rate, body mass index, smoking, drinking, hypertension, diabetes, cardiovascular disease, chronic kidney disease,poverty income ratio dummy variable, fasting blood glucose dummy variable, hemoglobin A1c dummy variable, triglyceride dummy variable, high-density lipoprotein dummy variable, low-density lipoprotein dummy variable, urinary albumin-to-creatinine ratio dummy variable, estimated glomerular filtration rate dummy variable, body mass index dummy variable, hypertension dummy variable, diabetes dummy variable, cardiovascular disease dummy variable,chronic kidney disease dummy variable, smoking dummy variable, drinking dummy variable.

**Supplement figure 2 U-shaped nonlinear relationship between log α-Klotho and all-cause mortality in different subgroups(I)**

**
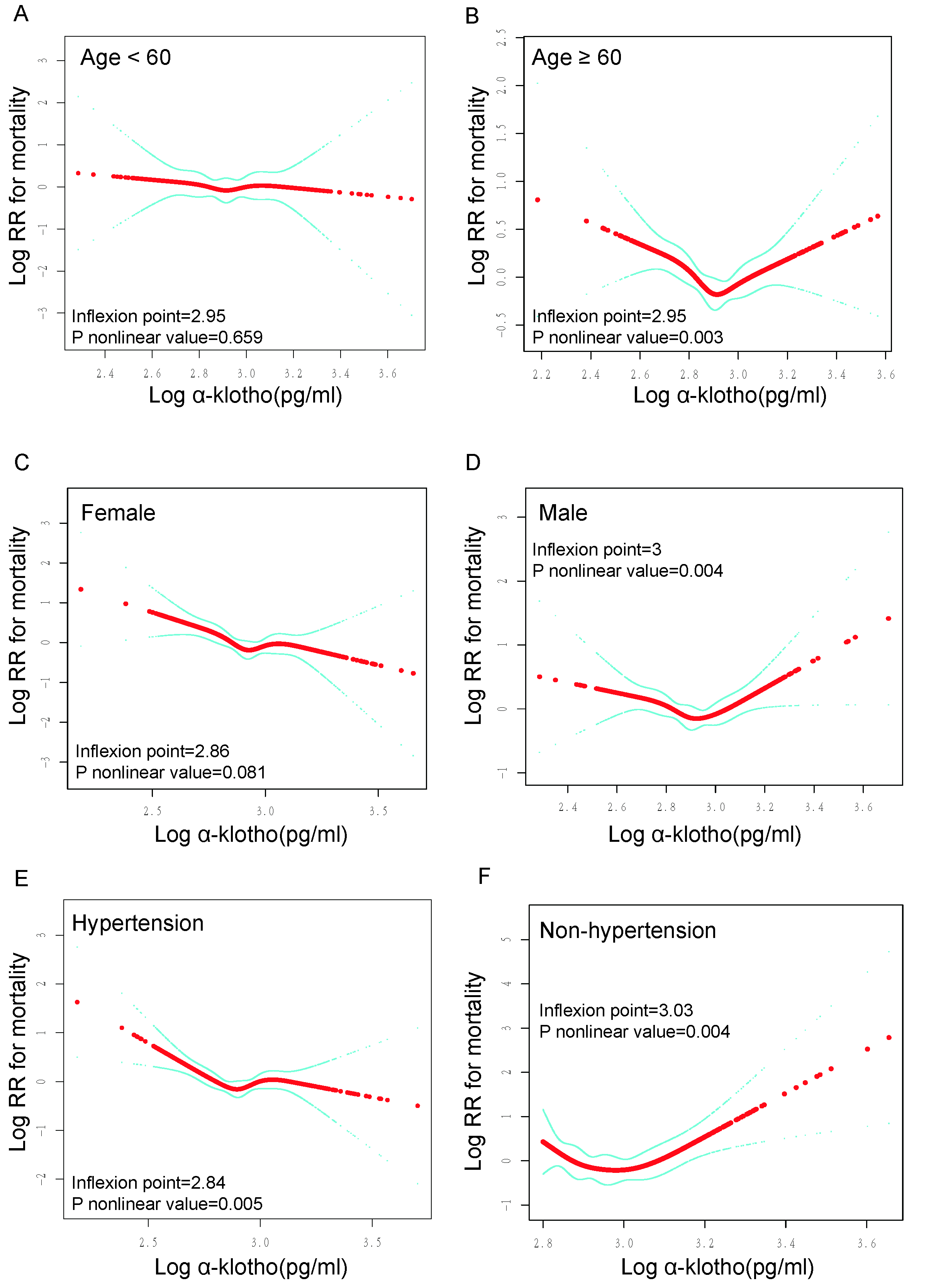
**

RR:relative risk.

Adjusted for age, sex, ethnic, poverty income ratio, education, fasting blood glucose, hemoglobin A1c, serum uric acid, triglyceride, total cholesterol, high-density lipoprotein, low-density lipoprotein, urinary albumin-to-creatinine ratio, estimated glomerular filtration rate, body mass index, smoking, drinking, hypertension, diabetes, cardiovascular disease, chronic kidney disease,poverty income ratio dummy variable, fasting blood glucose dummy variable, hemoglobin A1c dummy variable, triglyceride dummy variable, high-density lipoprotein dummy variable, low-density lipoprotein dummy variable, urinary albumin-to-creatinine ratio dummy variable, estimated glomerular filtration rate dummy cardiovascular disease dummy variable,chronic kidney disease dummy variable, smoking dummy variable, drinking dummy variable, except for themselves.

**Supplement figure 3 U-shaped nonlinear relationship between log α-Klotho and all-cause mortality in different subgroups(II)**

**
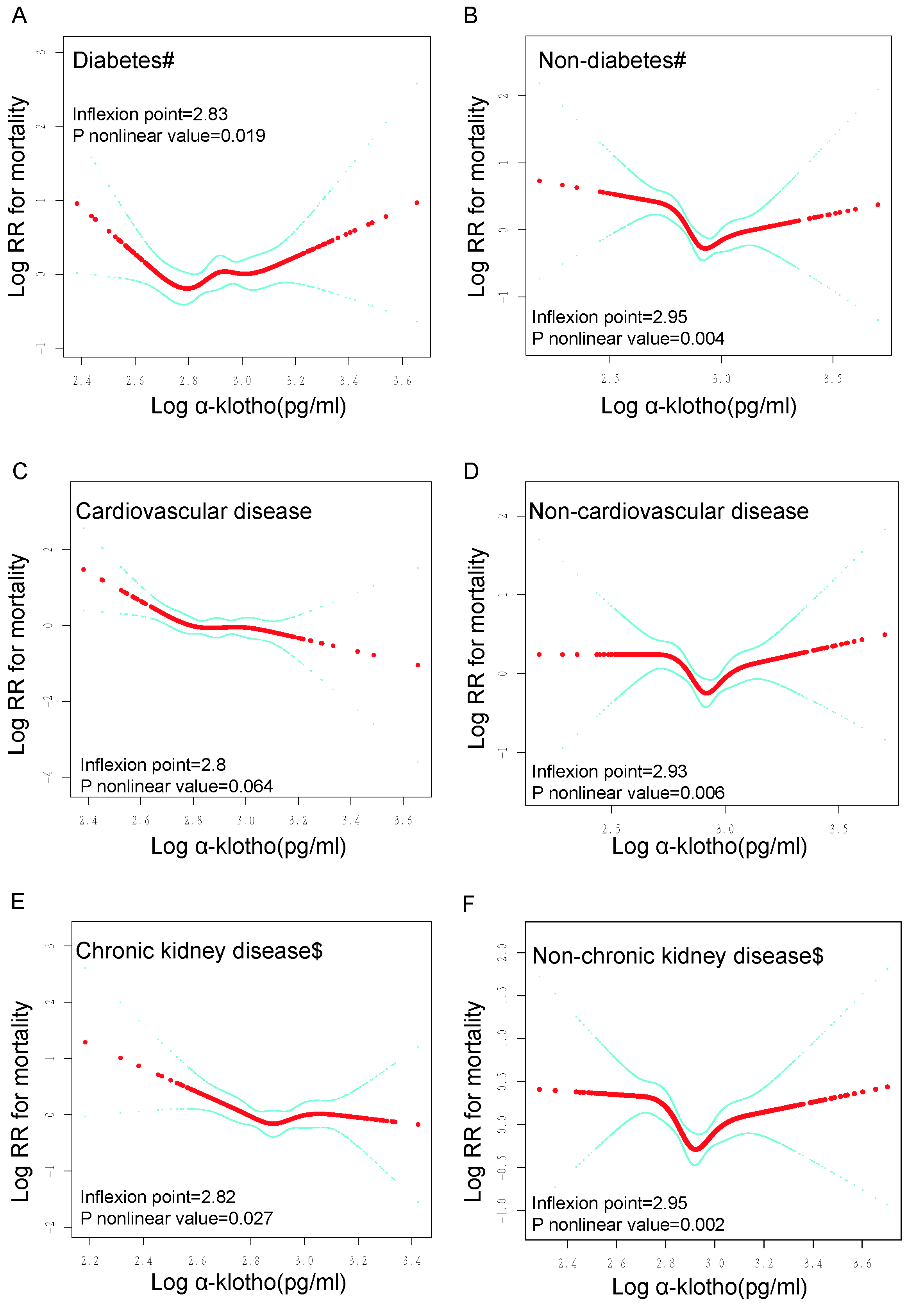
**

RR:relative risk.

Adjusted for age, sex, ethnic, poverty income ratio, education, fasting blood glucose, hemoglobin A1c, serum uric acid, triglyceride, total cholesterol, high-density lipoprotein, low-density lipoprotein, urinary albumin-to-creatinine ratio, estimated glomerular filtration rate, body mass index, smoking, drinking, hypertension, diabetes, cardiovascular disease, chronic kidney disease,poverty income ratio dummy variable, fasting blood glucose dummy variable, hemoglobin A1c dummy variable, triglyceride dummy variable, high-density lipoprotein dummy variable, low-density lipoprotein dummy variable, urinary albumin-to-creatinine ratio dummy variable, estimated glomerular filtration rate dummy variable, body mass index dummy variable, hypertension dummy variable, diabetes dummy variable, cardiovascular disease dummy variable,chronic kidney disease dummy variable, smoking dummy variable, drinking dummy variable, except for themselves. Unless specifically marked.

#:Adjusted for age, sex, ethnic, poverty income ratio, education, serum uric acid, triglyceride, total cholesterol, high-density lipoprotein, low-density lipoprotein, urinary albumin-to-creatinine ratio, estimated glomerular filtration rate, body mass index, smoking, drinking, hypertension, cardiovascular disease, chronic kidney disease,poverty income ratio dummy variable, triglyceride dummy variable, high-density lipoprotein dummy variable, low-density lipoprotein dummy variable, urinary albumin-to-creatinine ratio dummy variable, estimated glomerular filtration rate dummy variable, body mass index dummy variable, hypertension dummy variable, cardiovascular disease dummy variable,chronic kidney disease dummy variable, smoking dummy variable, drinking dummy variable.

$:Adjusted for age, sex, ethnic, poverty income ratio, education, fasting blood glucose, hemoglobin A1c, serum uric acid, triglyceride, total cholesterol, high-density lipoprotein, low-density lipoprotein, body mass index, smoking, drinking, hypertension, diabetes, cardiovascular disease, poverty income ratio dummy variable, fasting blood glucose dummy variable, hemoglobin A1c dummy variable, triglyceride dummy variable, high-density lipoprotein dummy variable, low-density lipoprotein dummy variable, body mass index dummy variable, hypertension dummy variable, diabetes dummy variable, cardiovascular disease dummy variable, smoking dummy variable, drinking dummy variable.
