# Supplementary material for: The inflection point: α-Klotho levels and the risk of all-cause mortality
Source: Front Endocrinol (Lausanne). 2025 Mar 11;16:1405003. doi: 10.3389/fendo.2025.1405003 (PMC11932894; doi:10.3389/fendo.2025.1405003)
Supplement: Supplementary file 2 [file Table1.doc]

**Supplement table 1 Dummy variables for variables containing missing values in NHANES 2007-2016**

| **Mortality** | No | Yes | P-value |
| --- | --- | --- | --- |
| **PIR (%)** |  |  | <0.001 |
| 0-1.39 | 3552 (29.16%) | 647 (41.24%) |  |
| 1.40-3.50 | 3670 (30.13%) | 540 (34.42%) |  |
| 3.51-5.00 | 3941 (32.36%) | 269 (17.14%) |  |
| Dummy variable | 1016 (8.34%) | 113 (7.20%) |  |
| **Education [N (%)]** |  |  | <0.001 |
| Junior high school education or below | 3301 (27.10%) | 586 (37.35%) |  |
| High school education | 2668 (21.91%) | 379 (24.16%) |  |
| College education or above | 6205 (50.95%) | 601 (38.30%) |  |
| Dummy variable | 5 (0.04%) | 3 (0.19%) |  |
| **FBG (mmol/L)** |  |  | <0.001 |
| 1.17-5.44 | 1919 (15.76%) | 178 (11.34%) |  |
| 5.45-6.16 | 2132 (17.51%) | 238 (15.17%) |  |
| 6.17-32.42 | 1886 (15.49%) | 357 (22.75%) |  |
| Dummy variable | 6242 (51.25%) | 796 (50.73%) |  |
| **HBA1C (%)** |  |  | <0.001 |
| 2.0-5.5 | 3862 (31.71%) | 405 (25.81%) |  |
| 5.6-5.9 | 4020 (33.01%) | 392 (24.98%) |  |
| 6.0-17.8 | 4279 (35.13%) | 770 (49.08%) |  |
| Dummy variable | 18 (0.15%) | 2 (0.13%) |  |
| **SUA (μmol/L)** |  |  | <0.001 |
| 23.8-285.5 | 3946 (32.40%) | 352 (22.43%) |  |
| 285.6-356.9 | 4085 (33.54%) | 471 (30.02%) |  |
| 357.0-1070.6 | 4142 (34.01%) | 745 (47.48%) |  |
| Dummy variable | 6 (0.05%) | 1 (0.06%) |  |
| **TG(mmol/L)** |  |  | 0.003 |
| 0.226-0.994 | 1970 (16.18%) | 230 (14.66%) |  |
| 0.995-1.581 | 1977 (16.23%) | 234 (14.91%) |  |
| 1.582-47.791 | 1930 (15.85%) | 303 (19.31%) |  |
| Dummy variable | 6302 (51.74%) | 802 (51.12%) |  |
| **TC(mmol/L)** |  |  | <0.001 |
| 1.94-4.63 | 3833 (31.47%) | 710 (45.25%) |  |
| 4.64-5.53 | 4164 (34.19%) | 425 (27.09%) |  |
| 5.54-21.02 | 4181 (34.33%) | 434 (27.66%) |  |
| Dummy variable | 1 (0.01%) | 0 (0.00%) |  |
| **HDL(mmol/L)** |  |  | <0.001 |
| 0.16-1.14 | 3671 (30.14%) | 599 (38.18%) |  |
| 1.15-1.47 | 4142 (34.01%) | 492 (31.36%) |  |
| 1.48-5.84 | 4365 (35.84%) | 478 (30.47%) |  |
| Dummy variable | 1 (0.01%) | 0 (0.00%) |  |
| **LDL(mmol/L)** |  |  | <0.001 |
| 0.388-2.612 | 1818 (14.93%) | 343 (21.86%) |  |
| 2.613-3.388 | 1968 (16.16%) | 195 (12.43%) |  |
| 3.389-8.560 | 1983 (16.28%) | 207 (13.19%) |  |
| Dummy variable | 6410 (52.63%) | 824 (52.52%) |  |
| **UACR (mg/g)** |  |  | <0.001 |
| 0.26-5.70 | 4237 (34.79%) | 288 (18.36%) |  |
| 5.71-11.30 | 4133 (33.94%) | 393 (25.05%) |  |
| 11.31-13787.89 | 3710 (30.46%) | 816 (52.01%) |  |
| Dummy variable | 99 (0.81%) | 72 (4.59%) |  |
| **eGFR(mL/min/1.73m^2^)** |  |  | <0.001 |
| 1.86-80.01 | 3712 (30.48%) | 869 (55.39%) |  |
| 80.02-97.28 | 4127 (33.89%) | 454 (28.94%) |  |
| 97.29-207.58 | 4335 (35.59%) | 246 (15.68%) |  |
| Dummy variable | 5 (0.04%) | 0 (0.00%) |  |
| **BMI (Kg/m^2^)** |  |  | <0.001 |
| 13.18-26.40 | 3960 (32.51%) | 529 (33.72%) |  |
| 26.41-31.35 | 4096 (33.63%) | 467 (29.76%) |  |
| 31.36-82.10 | 4019 (33.00%) | 510 (32.50%) |  |
| Dummy variable | 104 (0.85%) | 63 (4.02%) |  |
| **Smoking [N (%)]** |  |  | <0.001 |
| No | 6533 (53.64%) | 532 (33.91%) |  |
| Yes | 5640 (46.31%) | 1036 (66.03%) |  |
| Dummy variable | 6 (0.05%) | 1 (0.06%) |  |
| **Drinking [N (%)]** |  |  | <0.001 |
| No | 1664 (13.66%) | 204 (13.00%) |  |
| Yes | 9614 (78.94%) | 1257 (80.11%) |  |
| Dummy variable | 901 (7.40%) | 108 (6.88%) |  |
| **Hypertension [N (%)]** |  |  | <0.001 |
| No | 5952 (48.87%) | 409 (26.07%) |  |
| Yes | 6226 (51.12%) | 1160 (73.93%) |  |
| Dummy variable | 1 (0.01%) | 0 (0.00%) |  |
| **Diabetes [N (%)]** |  |  | <0.001 |
| No | 9380 (77.02%) | 930 (59.27%) |  |
| Yes | 2791 (22.92%) | 638 (40.66%) |  |
| Dummy variable | 8 (0.07%) | 1 (0.06%) |  |
| **CVD[N (%)]** |  |  | <0.001 |
| No | 10822 (88.86%) | 1019 (64.95%) |  |
| Yes | 1355 (11.13%) | 550 (35.05%) |  |
| Dummy variable | 2 (0.02%) | 0 (0.00%) |  |
| **CKD[N (%)]** |  |  | <0.001 |
| No | 10016 (82.24%) | 826 (52.64%) |  |
| Yes | 2095 (17.20%) | 703 (44.81%) |  |
| Dummy variable | 68 (0.56%) | 40 (2.55%) |  |

Continuous variables are expressed using medians (Q1-Q3) and categorical variables are expressed using percentages.

PIR: poverty income ratio; FBG: fasting blood glucose; HbA1C: hemoglobin A1c; SUA: serum uric acid; TG: triglyceride; TC: total cholesterol; HDL: high-density lipoprotein; LDL: low-density lipoprotein; UACR: urinary albumin-to-creatinine ratio; eGFR: estimated glomerular filtration rate; BMI: body mass index; CVD: cardiovascular disease; CKD: chronic kidney disease.

**Supplement table 2 Univariate Cox regression for variables associated with all-cause mortality outcomes**

| Variables | Mortality  (HR 95%CI) | P Value |
| --- | --- | --- |
| **Age** | 1.09 (1.08, 1.09) | <0.0001 |
| **Sex** |  |  |
| Female | 1.0 |  |
| Male | 1.55 (1.40, 1.71) | <0.0001 |
| **Ethnic [N (%)]** |  |  |
| Non-Hispanic white | 1.0 |  |
| Non-Hispanic black | 1.85 (1.53, 2.23) | <0.0001 |
| Mexican American | 1.90 (1.61, 2.25) | <0.0001 |
| Other | 1.07 (0.87, 1.32) | 0.5015 |
| **Education [N (%)]** |  |  |
| Junior high school education or below | 1.0 |  |
| High school education | 0.83 (0.73, 0.95) | 0.0056 |
| College education or above | 0.61 (0.54, 0.68) | <0.0001 |
| **PIR (%)** | 0.79 (0.76, 0.81) | <0.0001 |
| **Log Klotho [(pg/ml)](http://dspp-hane-1601/Nchs/Edit/DocumentationView.aspx?Id=7612&Dataset=SSKL_E#SSKLOTH)** | 1.00 (0.99, 1.00) | <0.0001 |
| **FBG (mmol/L)** | 1.09 (1.07, 1.11) | <0.0001 |
| **HBA1C (%)** | 1.18 (1.14, 1.21) | <0.0001 |
| **SUA (μmol/L)** | 1.00 (1.00, 1.00) | <0.0001 |
| **TG (mmol/L)** | 1.02 (0.98, 1.06) | 0.3415 |
| **TC (mmol/L)** | 0.76 (0.72, 0.80) | <0.0001 |
| **HDL (mmol/L)** | 0.80 (0.71, 0.90) | 0.0003 |
| **LDL (mmol/L)** | 0.68 (0.62, 0.73) | <0.0001 |
| **UACR (mg/g)** | 1.00 (1.00, 1.00) | <0.0001 |
| **eGFR(mL/min/1.73m^2^)** | 0.97 (0.97, 0.97) | <0.0001 |
| **BMI (Kg/m^2^)** | 1.00 (1.00, 1.01) | 0.3829 |
| **Smoking [N (%)]** |  |  |
| No | 1.0 |  |
| Yes | 2.06 (1.86, 2.29) | <0.0001 |
| **Drinking [N (%)]** |  |  |
| No | 1.0 |  |
| Now | 0.77 (0.66, 0.90) | 0.0009 |
| Former | 1.70 (1.45, 2.00) | <0.0001 |
| **Hypertension [N (%)]** |  |  |
| No | 1.0 |  |
| Yes | 2.59 (2.32, 2.90) | <0.0001 |
| **Diabetes [N (%)]** |  |  |
| No | 1.0 |  |
| Yes | 2.26 (2.05, 2.50) | <0.0001 |
| **CVD [N (%)]** |  |  |
| No | 1.0 |  |
| Yes | 3.80 (3.42, 4.21) | <0.0001 |
| **CKD [N (%)]** |  |  |
| No | 1.0 |  |
| Yes | 3.67 (3.32, 4.05) | <0.0001 |

PIR: poverty income ratio; FBG: fasting blood glucose; HbA1C: hemoglobin A1c; SUA: serum uric acid; TG: triglyceride; TC: total cholesterol; HDL: high-density lipoprotein; LDL: low-density lipoprotein; UACR: urinary albumin-to-creatinine ratio; eGFR: estimated glomerular filtration rate; BMI: body mass index; CVD: cardiovascular disease; CKD: chronic kidney disease.

**Supplement table 3 Multivariate Cox proportional risk regression analysis of log klotho in relation to all-cause mortality in NHANES 2007-2016**

| **Exposure** | **Crude** | **Model 1** | **Model 2** | **Model 3** |
| --- | --- | --- | --- | --- |
| **Log Klotho [(pg/ml)](http://dspp-hane-1601/Nchs/Edit/DocumentationView.aspx?Id=7612&Dataset=SSKL_E#SSKLOTH)** | 0.34 (0.24, 0.48) <0.0001 | 0.67 (0.47, 0.95) 0.0254 | 0.57 (0.32, 1.01) 0.0522 | 0.56 (0.31, 0.99) 0.0473 |
| **Log Klotho quartile (pg/ml)** |  |  |  |  |
| Quartile 1  2.18-2.81 | 0.15 (0.06, 0.41) 0.0002 | 0.27 (0.10, 0.72) 0.0090 | 0.29 (0.05, 1.71) 0.1702 | 0.15 (0.02, 0.96) 0.0455 |
| Quartile 2  2.82-2.90 | 0.03 (0.00, 1.50) 0.0775 | 0.11 (0.00, 6.66) 0.2881 | 0.08 (0.00, 59.42) 0.4545 | 0.07 (0.00, 69.38) 0.4526 |
| Quartile 3  2.91-2.99 | 0.04 (0.00, 2.12) 0.1115 | 0.03 (0.00, 2.35) 0.1181 | 0.02 (0.00, 19.38) 0.2631 | 0.03 (0.00, 37.42) 0.3289 |
| Quartile 4  3.0-3.70 | 5.96 (2.02, 17.60) 0.0012 | 9.61 (3.13, 29.47) <0.0001 | 5.39 (0.90, 32.31) 0.0655 | 3.92 (0.65, 23.79) 0.1372 |
| **Survival time (years)** |  |  |  |  |
| <5 | 0.54 (0.33, 0.89) 0.0154 | 1.09 (0.65, 1.82) 0.7470 | 0.92 (0.40, 2.13) 0.8533 | 1.02 (0.43, 2.40) 0.9633 |
| 5-10 | 0.42 (0.25, 0.71) 0.0010 | 0.79 (0.45, 1.36) 0.3884 | 0.57 (0.24, 1.32) 0.1879 | 0.59 (0.25, 1.38) 0.2204 |
| >10 | 0.84 (0.27, 2.63) 0.7659 | 1.82 (0.55, 6.06) 0.3298 | 1.89 (0.21, 16.80) 0.5676 | 1.79 (0.16, 19.79) 0.6347 |

Data are presented as HR (95% CI) and P value unless indicated otherwise.

Model 1: Adjusted for age; sex; Ethnic; poverty income ratio; Education.

Model 2: Adjusted for Model 1+fasting blood glucose; hemoglobin A1c; serum uric acid; triglyceride; total cholesterol; high-density lipoprotein; low-density lipoprotein; urinary albumin-to-creatinine ratio; estimated glomerular filtration rate; body mass index; smoking; drinking; hypertension; diabetes; cardiovascular disease; chronic kidney disease.

Model 3: Adjusted for Model 2+poverty income ratio dummy variable; fasting blood glucose dummy variable; hemoglobin A1c dummy variable, triglyceride dummy variable, high-density lipoprotein dummy variable, low-density lipoprotein dummy variable, urinary albumin-to-creatinine ratio dummy variable, estimated glomerular filtration rate dummy variable, body mass index dummy variable, hypertension dummy variable, diabetes dummy variable, cardiovascular disease dummy variable,chronic kidney disease dummy variable; smoking dummy variable, drinking dummy variable.

**Supplement table 4 Two-piecewise cox proportional risk regression analysis of the effect of all-cause mortality in NHANES, 2007-2016 According to different subgroups.**

| Outcome: | All-cause mortality | | |
| --- | --- | --- | --- |
|  | HR (95%CI) | P Value | P nonlinear value (P for log-likelihood ratio test) |
| Age < 60 |  |  |  |
| Log klotho < 2.95 pg/ml | 0.54 (0.08, 3.56) | 0.5232 | 0.659 |
| Log klotho > 2.95 pg/ml | 1.24 (0.10, 14.99) | 0.8632 |  |
| Age ≥ 60 |  |  |  |
| Log klotho< 2.95 pg/ml | 0.21 (0.08, 0.56) | 0.0016 | 0.003 |
| Log klotho < 2.95 pg/ml | 4.63 (1.17, 18.29) | 0.0287 |  |
| Female |  |  |  |
| Log klotho < 2.86 pg/ml | 0.07 (0.01, 0.39) | 0.0023 | 0.081 |
| Log klotho < 2.86 pg/ml | 0.79 (0.19, 3.35) | 0.7523 |  |
| Male |  |  |  |
| Log klotho < 3 pg/ml | 0.34 (0.13, 0.90) | 0.0294 | 0.004 |
| Log klotho < 3 pg/ml | 1.39 (0.50, 3.84) | 0.5239 |  |
| Hypertension |  |  |  |
| Log klotho < 2.84 pg/ml | 0.07 (0.02, 0.28) | 0.0002 | 0.005 |
| Log klotho < 2.84 pg/ml | 13.04 (2.23, 76.36) | 0.0044 |  |
| Non-hypertension |  |  |  |
| Log klotho < 3.03 pg/ml | 0.24 (0.06, 1.01) | 0.0510 | 0.004 |
| Log klotho < 3.03 pg/ml | 80.18 (4.66, 1378.59) | 0.0025 |  |
| Diabetes^#^ |  |  |  |
| Log klotho < 2.83 pg/ml | 0.14 (0.02, 0.98) | 0.0477 | 0.019 |
| Log klotho < 2.83 pg/ml | 3.92 (1.16, 13.28) | 0.0282 |  |
| Non-diabetes^#^ |  |  |  |
| Log klotho < 2.95 pg/ml | 0.09 (0.03, 0.27) | <0.0001 | 0.004 |
| Log klotho < 2.95 pg/ml | 3.02 (0.59, 15.40) | 0.1836 |  |
| Cardiovascular disease |  |  |  |
| Log klotho < 2.8 pg/ml | 0.03 (0.00, 0.34) | 0.0047 | 0.064 |
| Log klotho < 2.8 pg/ml | 0.72 (0.17, 2.99) | 0.6483 |  |
| Non-cardiovascular disease |  |  |  |
| Log klotho < 2.93 pg/ml | 0.20 (0.06, 0.61) | 0.0048 | 0.006 |
| Log klotho < 2.93 pg/ml | 3.57 (1.01, 12.69) | 0.0488 |  |
| Chronic kidney disease^$^ |  |  |  |
| Log klotho < 2.82 pg/ml | 0.08 (0.01, 0.44) | 0.0041 | 0.027 |
| Log klotho < 2.82 pg/ml | 1.55 (0.40, 5.93) | 0.5226 |  |
| Non-chronic kidney disease^$^ |  |  |  |
| Log klotho < 2.95 pg/ml | 0.13 (0.04, 0.41) | 0.0005 | 0.002 |
| Log klotho < 2.95 pg/ml | 4.37 (1.10, 17.46) | 0.0367 |  |

Data are adjusted for age, sex, ethnic, poverty income ratio, education, fasting blood glucose, hemoglobin A1c, serum uric acid, triglyceride, total cholesterol, high-density lipoprotein, low-density lipoprotein, urinary albumin-to-creatinine ratio, estimated glomerular filtration rate, body mass index, smoking, drinking, hypertension, diabetes, cardiovascular disease, chronic kidney disease,poverty income ratio dummy variable, fasting blood glucose dummy variable, hemoglobin A1c dummy variable, triglyceride dummy variable, high-density lipoprotein dummy variable, low-density lipoprotein dummy variable, urinary albumin-to-creatinine ratio dummy variable, estimated glomerular filtration rate dummy variable, body mass index dummy variable, hypertension dummy variable, diabetes dummy variable, cardiovascular disease dummy variable,chronic kidney disease dummy variable, smoking dummy variable, drinking dummy variable, except for themselves. Unless specifically marked.

#:Data are adjusted for age, sex, ethnic, poverty income ratio, education, serum uric acid, triglyceride, total cholesterol, high-density lipoprotein, low-density lipoprotein, urinary albumin-to-creatinine ratio, estimated glomerular filtration rate, body mass index, smoking, drinking, hypertension, cardiovascular disease, chronic kidney disease,poverty income ratio dummy variable, triglyceride dummy variable, high-density lipoprotein dummy variable, low-density lipoprotein dummy variable, urinary albumin-to-creatinine ratio dummy variable, estimated glomerular filtration rate dummy variable, body mass index dummy variable, hypertension dummy variable, cardiovascular disease dummy variable,chronic kidney disease dummy variable, smoking dummy variable, drinking dummy variable.

$:Data are adjusted for age, sex, ethnic, poverty income ratio, education, fasting blood glucose, hemoglobin A1c, serum uric acid, triglyceride, total cholesterol, high-density lipoprotein, low-density lipoprotein, body mass index, smoking, drinking, hypertension, diabetes, cardiovascular disease, poverty income ratio dummy variable, fasting blood glucose dummy variable, hemoglobin A1c dummy variable, triglyceride dummy variable, high-density lipoprotein dummy variable, low-density lipoprotein dummy variable, body mass index dummy variable, hypertension dummy variable, diabetes dummy variable, cardiovascular disease dummy variable, smoking dummy variable, drinking dummy variable.
